# The University of Pennsylvania glioblastoma (UPenn-GBM) cohort: advanced MRI, clinical, genomics, & radiomics

**DOI:** 10.1038/s41597-022-01560-7

**Published:** 2022-07-29

**Authors:** Spyridon Bakas, Chiharu Sako, Hamed Akbari, Michel Bilello, Aristeidis Sotiras, Gaurav Shukla, Jeffrey D. Rudie, Natali Flores Santamaría, Anahita Fathi Kazerooni, Sarthak Pati, Saima Rathore, Elizabeth Mamourian, Sung Min Ha, William Parker, Jimit Doshi, Ujjwal Baid, Mark Bergman, Zev A. Binder, Ragini Verma, Robert A. Lustig, Arati S. Desai, Stephen J. Bagley, Zissimos Mourelatos, Jennifer Morrissette, Christopher D. Watt, Steven Brem, Ronald L. Wolf, Elias R. Melhem, MacLean P. Nasrallah, Suyash Mohan, Donald M. O’Rourke, Christos Davatzikos

**Affiliations:** 1 Center for Biomedical Image Computing and Analytics (CBICA), University of Pennsylvania, Philadelphia, PA USA; 2 Department of Radiology, Perelman School of Medicine, University of Pennsylvania, Philadelphia, PA USA; 3 Department of Pathology and Laboratory Medicine, Perelman School of Medicine, University of Pennsylvania, Philadelphia, PA USA; 4 Department of Radiology and Institute for Informatics, Washington University, School of Medicine, St. Louis, MO USA; 5 Department of Radiation Oncology, Christiana Care Health System, Philadelphia, PA USA; 6 Department of Radiology & Biomedical Imaging, University of California, San Francisco, San Francisco, CA USA; 7 Department of Neurosurgery, Perelman School of Medicine, University of Pennsylvania, Philadelphia, PA USA; 8 Department of Radiation Oncology, Perelman School of Medicine, University of Pennsylvania, Philadelphia, PA USA; 9 Division of Hematology Oncology, Perelman School of Medicine, University of Pennsylvania, Philadelphia, PA USA; 10 Department of Diagnostic Radiology and Nuclear Medicine, University of Maryland School of Medicine, Baltimore, MD USA

**Keywords:** CNS cancer, Translational research, Biomarkers, Magnetic resonance imaging, Research data

## Abstract

Glioblastoma is the most common aggressive adult brain tumor. Numerous studies have reported results from either private institutional data or publicly available datasets. However, current public datasets are limited in terms of: a) number of subjects, b) lack of consistent acquisition protocol, c) data quality, or d) accompanying clinical, demographic, and molecular information. Toward alleviating these limitations, we contribute the “University of Pennsylvania Glioblastoma Imaging, Genomics, and Radiomics” (UPenn-GBM) dataset, which describes the currently largest publicly available comprehensive collection of 630 patients diagnosed with *de novo* glioblastoma. The UPenn-GBM dataset includes (a) advanced multi-parametric magnetic resonance imaging scans acquired during routine clinical practice, at the University of Pennsylvania Health System, (b) accompanying clinical, demographic, and molecular information, (d) perfusion and diffusion derivative volumes, (e) computationally-derived and manually-revised expert annotations of tumor sub-regions, as well as (f) quantitative imaging (also known as radiomic) features corresponding to each of these regions. This collection describes our contribution towards repeatable, reproducible, and comparative quantitative studies leading to new predictive, prognostic, and diagnostic assessments.

## Abstract (summary)

**Table:** 

Measurement(s)	Magnetic Resonance Imaging
Technology Type(s)	Magnetic Resonance Imaging of the Brain with and without Contrast
Sample Characteristic - Organism	Homo sapiens
Sample Characteristic - Environment	brain
Sample Characteristic - Location	United States of America

## Background & Summary

Glioblastoma (GBM) is the most common, complex, and aggressive adult primary tumor of the central nervous system (CNS). Although the currently applicable standard-of-care treatment options (i.e., surgery, radiotherapy, chemotherapy) have expanded during the last 20 years, there is no substantial improvement in patient overall survival (OS). Despite various attempts targeting diagnostic and therapeutic advances, the reported prognostication of GBM patients still remains at a median OS rate of 16–20 months following standard of care therapy and 5-year survival rate of 10%^1^. A major obstacle in treating GBM and extending patient OS relates, in part, to the underlying spatio-temporal heterogeneity of its molecular and micro-environmental landscape that are also reflected at the phenotypic level^2–14^. Numerous translational, computational, and clinical research studies have been conducted and reported results from either private institutional data or publicly available datasets^15–30^. However, current public datasets are limited in terms of: (a) the number of included subjects, (b) lack of consistent acquisition protocol, (c) variable quality of data, or (d) accompanying clinical, demographic, and molecular information.

**Fig. 1:**
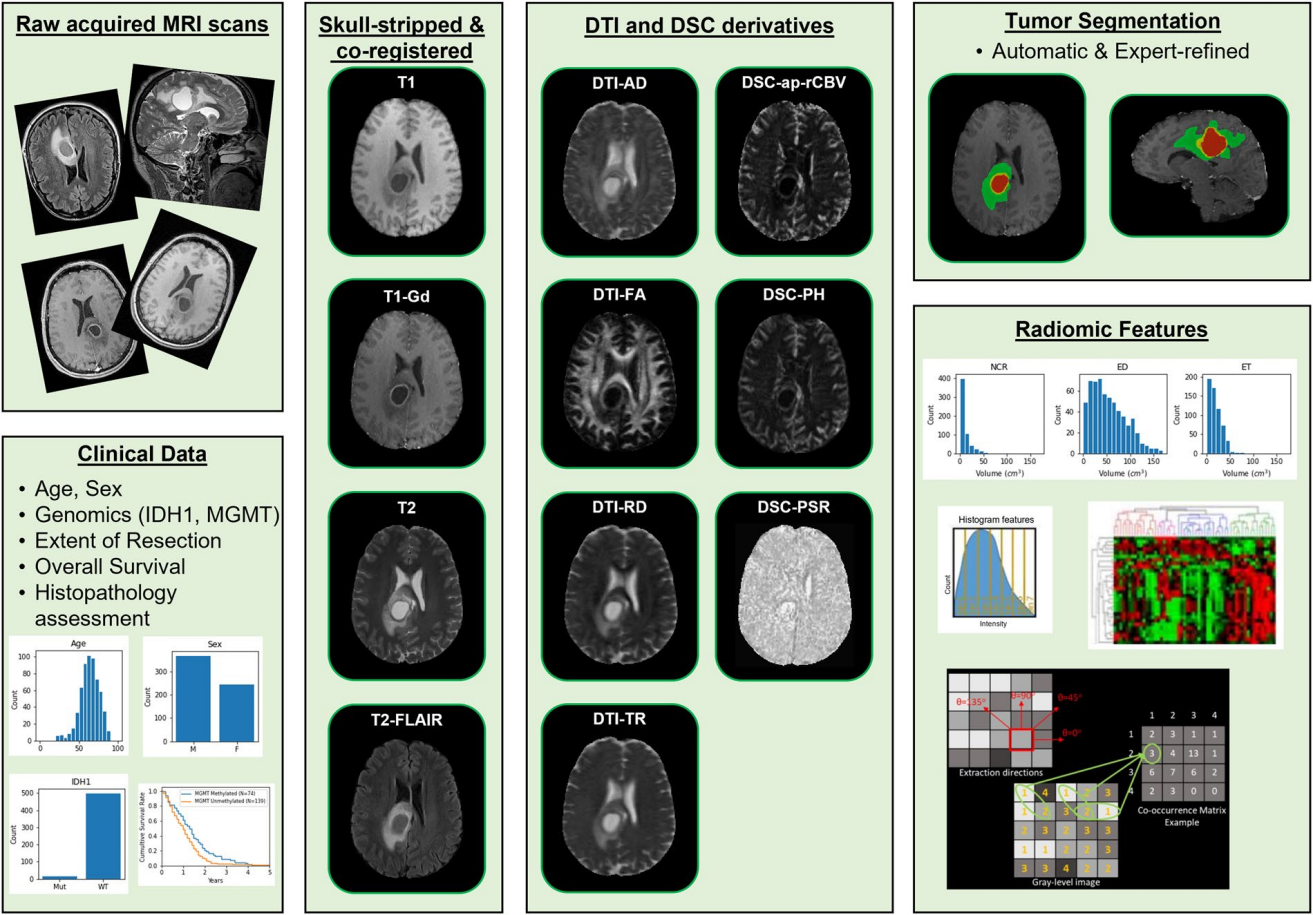
Visual summary of the “University of Pennsylvania Glioblastoma Advanced Imaging, Clinical, Genomics, and Radiomics” (UPenn-GBM) data collection.

To address these limitations, and facilitate further studies towards understanding mechanisms of this disease, we introduce the “University of Pennsylvania Glioblastoma Imaging, Genomics, and Radiomics” (UPenn-GBM) dataset, which describes the currently largest publicly available comprehensive dataset of 630 patients diagnosed with *de novo* GBM (Fig. 1). The complete UPenn-GBM collection is made freely available to browse, download, and use via The Cancer Imaging Archive (TCIA)^31^, as outlined in the Creative Commons Attribution Unported (CC BY) License.

The UPenn-GBM collection specifically includes a) advanced multi-parametric magnetic resonance imaging (mpMRI) scans acquired consistently at the University of Pennsylvania Health System (UPHS) during routine clinical radiologic exams, at the pre-operative baseline time-point for 611 patients, and at the follow up time-point prior to second surgery for a subset of them, b) accompanying clinical and demographic data, inclusive of age, gender, resection status, Karnofsky performance score (KPS) prior to treatment, survival information from first surgical operation, and a predicted pseudoprogression index, c) molecular status for Isocitrate dehydrogenase 1 (IDH1) mutations based on next generation sequencing (NGS) and/or immunohistochemical staining for IDH1-R132H, and methylation of the O(6)-Methylguanine-DNA methyltransferase (MGMT) promoter based on pyrosequencing^32^. In addition to these clinically acquired data, the UPenn-GBM dataset further includes: d) pre-processed scans according to a standardized protocol^15–18,33^ (inclusive of co-registration of all mpMRI scans in a common anatomical atlas, resampling to an isotropic resolution of 1*mm*^3^, and skull-stripping) e) extracted perfusion and diffusion derivative volumetric scans, f) computationally-derived and manually revised expert annotations of tumor sub-region boundaries, as well as g) quantitative imaging (also known as radiomic) features^34–36^, corresponding to each of these regions.

The contribution on the UPenn-GBM dataset is two-fold: (a) its potential for re-use towards repeatable, reproducible, and comparative quantitative translational, computational, and clinical research studies leading to new predictive, prognostic, and diagnostic assessments, enabled by direct utilization through TCIA^37^, and (b) benefiting both clinical and computational imaging researchers targeting the development and evaluation of quantitative algorithms for segmentation and downstream radiomic analyses associated with clinically relevant goals. Future planned extensions of this dataset will support more comprehensive radiogenomic studies, by incorporating most clinically-relevant molecular markers reported in the literature, as well as radio-patho-genomic research^38^ aiming to find phenotypic signatures of GBM molecular characteristics.

## Methods

In favor of transparency, in this section we provide a detailed description of all the procedures followed to produce the data of the UPenn-GBM collection, including all descriptions of the experimental design, data acquisition assays, and any computational processing (e.g., curation, brain extraction, tumor segmentation, radiomic feature extraction) towards enabling reproducible research.

### Data

The study population was identified on the basis of retrospective review of the electronic medical records of patients diagnosed with GBM at the UPHS from 2006 to 2018. 630 patients were included in the UPenn-GBM collection, 611 of which were selected according to the inclusion criteria of: (i) age ≥18 years old, and (ii) preoperative scans comprising baseline mpMRI at time of diagnosis, comprising the four structural MRI scans, i.e., native T1-weighted (T1), post-contrast T1 (T1-Gd), native T2-weighted (T2), and T2 fluid attenuated inversion recovery (T2-FLAIR) scans. Diffusion tensor imaging (DTI), and dynamic susceptibility contrast (DSC) MRI scans were also acquired and provided for most cases. In addition to the 611 baseline pre-operative scans, the UPenn-GBM collection includes 60 follow-up scans from patients who have undergone a second resection due to progressive radiographic changes. Notably 19 of these 60 cases had available data only the follow time-point. Summary demographics can be found in Table 1. A summary of the scanner manufacturer, scanner models, and acquisition settings used to capture each of the cases included in the UPenn-GBM data collection described here can be found and downloaded from the TCIA repository^37^.

**Table 1:** Demographics of the UPenn-GBM data collection.

Demographics	Value	Number	%
Gender	Female	252	40.0%
	Male	378	60.0%
Age (years)	18–29	14	2.2%
	30–49	69	11.0%
	50–69	367	58.3%
	70+	180	28.6%
Resection Status	Gross Total	362	59.2%
	Partial	211	34.5%
	Unknown	38	6.2%
Imaging	Structural scans	671	100.0%
	DTI	592	88.2%
	DSC	534	79.6%
Scan Time-point	Pre-operative	611	91.1%
	Follow up	60	8.9%
MGMT methylation status	Methylated	140	22.9%
	Unmethylated	177	29.0%
	Unknown	294	48.1%
IDH	Mutated	16	2.6%
	Wildtype	499	81.7%
	NOS/NEC	96	15.7%

Patients included in our study were treated according to standard of care, which included maximal safe resection, radiotherapy, and concomitant and adjuvant chemotherapy with Temolozolomide (TMZ). Collection, analysis, and release of the UPenn-GBM data has happened in compliance with all relevant ethical regulations. The protocol was approved by the Institutional Review Board at the UPHS, and informed consent was obtained from all participants.

### Clinical data

Clinico-pathologic information including age, sex, histologic diagnosis, and molecular data, if available, were obtained directly from the corresponding patient medical records. The age range of the included population was 18–89. The ratio of male:female was equal to 60:40. The resection status of the 611 patients with available pre-operative baseline scans was partitioned in the three categorical entries of i) Gross Total Resection (GTR, n = 362), ii) Partial Resection (PR, n = 211), and iii) Not Available (NA, *n* = 38), representing excision of ≥90%, <90%, and unknown proportion of the tumor, respectively. The Karnofsky performance score (KPS) prior to treatment was identified for 75 of the 611 baseline pre-operative scans. Overall survival data is provided for 452 patients, and additional data related to patient prognosis include IDH1 status and MGMT promoter methylation status (Table 1). All these are downloadable from TCIA^37^.

Clinico-pathologic information for the follow up cases include a pathological assessment score, in the range of 1–6, which indicates the degree of tumor progression and treatment effects, following expert evaluation of the related tissue sections (1 = <10%, 2 = 10%–25%, 3 = 25%–50%, 4 = 50%–75%, 5 = 75%–90%, and 6 = >90% malignant features). The exact inclusion criteria for these (as also described in the original study^39^ obtaining these scores), comprised 1) initial gross total resection of the tumor core followed by chemo-radiation, 2) new or increasing enhancement on follow-up MRI, 3) second resection and histopathological tissue evaluation, and 4) acquisition of all modalities (T1, T1-Gd, T2, T2-FLAIR, DSC, and DTI), within 15 days prior to the second resection.

### Molecular characterization

Following the current World Health Organization (WHO) classification of CNS tumors^40^, we focus on the mutational status of IDH that was identified for 515 of all the 611 cases with baseline pre-operative scans. The remaining 96 cases were classified according to “The Consortium to Inform Molecular and Practical Approaches to CNS Tumor Taxonomy–Not Official WHO (cIMPACT-NOW)” as IDH-Not-Otherwise-Specified (IDH-NOS)^41–48^. Note that the role of the cIMPACT-NOW board is to provide evidence-based scientific updates/refinements beyond the most recent WHO classification of CNS tumors^40^ towards ensuring the best possible clinical care of patients. Mutations in IDH were found for 16 of the 515 cases (3.11%), and the remaining ones were classified as wildtype IDH (IDHwt). The mutational status of IDH1 was determined by Next Generation Sequencing (NGS)^32^ and/or immunohistochemical staining for IDH1-R132H. Cases were sequenced on one of the two UPHS clinical solid tumor NGS panels^32^ or on a research NGS panel. The original 47-gene clinical panel used the TruSeq Custom Amplicon Cancer Panel kit (Illumina, San Diego, CA), which targeted hotspot variants. Samples were multiplexed and sequenced on a MiSeq to an average depth of coverage of 2500 ×. Subsequently, a larger panel was implemented with full gene coverage of 153 genes, using the Agilent Haloplex design with unique molecular identifiers. Samples were multiplexed and sequenced on a HiSeq with total deduplicated reads of 6.5 million reads/sample. For both clinical panels, variants were identified using an in-house data processing bioinformatics pipeline. For the research panel, libraries were prepared using a custom AmpliSeq panel, Ion AmpliSeq Library Kit 2.0 and templated on an Ion Torrent OneTouch 2 instrument. Templated libraries are enriched on an Ion OneTouch ES.

The MGMT promoter methylation status by pyrosequencing is available for 317 of the 611 cases, of which 140 had methylation detected and 177 had methylation not detected. To determine the MGMT promoter methylation status, genomic DNA was extracted from 5-micron tissue sections of formalin-fixed paraffin-embedded (FFPE) tissue samples containing at least 20% tumor cellularity. Approximately 500–1000 ng total DNA was subjected to bisulfite conversion using the EZ DNA Methylation Kit (Zymo Research, Irvine, CA). A total of 50–100 ng bisulfite-treated DNA was carried on for PCR using F-primer (50-GTTTYGGATATGTTGGGATA-30) and R-primer (50-biotin-ACCCAAACACTCACCAAATC-30), creating a fragment spanning 4 CpG sites in exon 1 of MGMT [(chr10:131,265,529-131,265,537; UCSC Genome Browser on Human Feb. 2009 (GRCh37/hg19) Assembly]. We then conducted the pyrosequencing methylation assay on the PyroMark Q24 (Qiagen) using the Pyromark MGMT kit to detect the ratio of T:C to determine the level of methylation at the 4 CpG sites. A mean and median percent methylation across all four CpG sites equal to or greater than 10% was interpreted as positive. A result with mean and median below 4.5% methylation was interpreted as negative. If both mean and median were greater than or equal to 4.5%, but at least one was less than 10%, then the result was designated low positive. If either the mean or median was less than 4.5% but the other was greater than or equal to 4.5%, then the result was designated indeterminate.

Note that both the IDH mutational status and the MGMT promoter methylation status were originally derived from the rigorously validated assays and procedures described above. Then the concluding labels were stored in the patients’ medical records as measurements used routinely for diagnostic and patient treatment purposes. The “UPenn-GBM” collection makes publicly available these concluding labels, as the underlying raw clinical data were not available for distribution.

### Image Pre-processing

Since the scans included in this study were heterogeneously obtained from different scanners and acquisition protocols, in addition to providing the scans in their original state (i.e., resolution, orientation), after de-identification and de-facing, they all underwent the same pre-processing protocol to make image dimensions and voxel sizes uniform across studies and modalities. Details of the original state of the acquired scans are provided at the TCIA repository^37^.

**Fig. 2:**
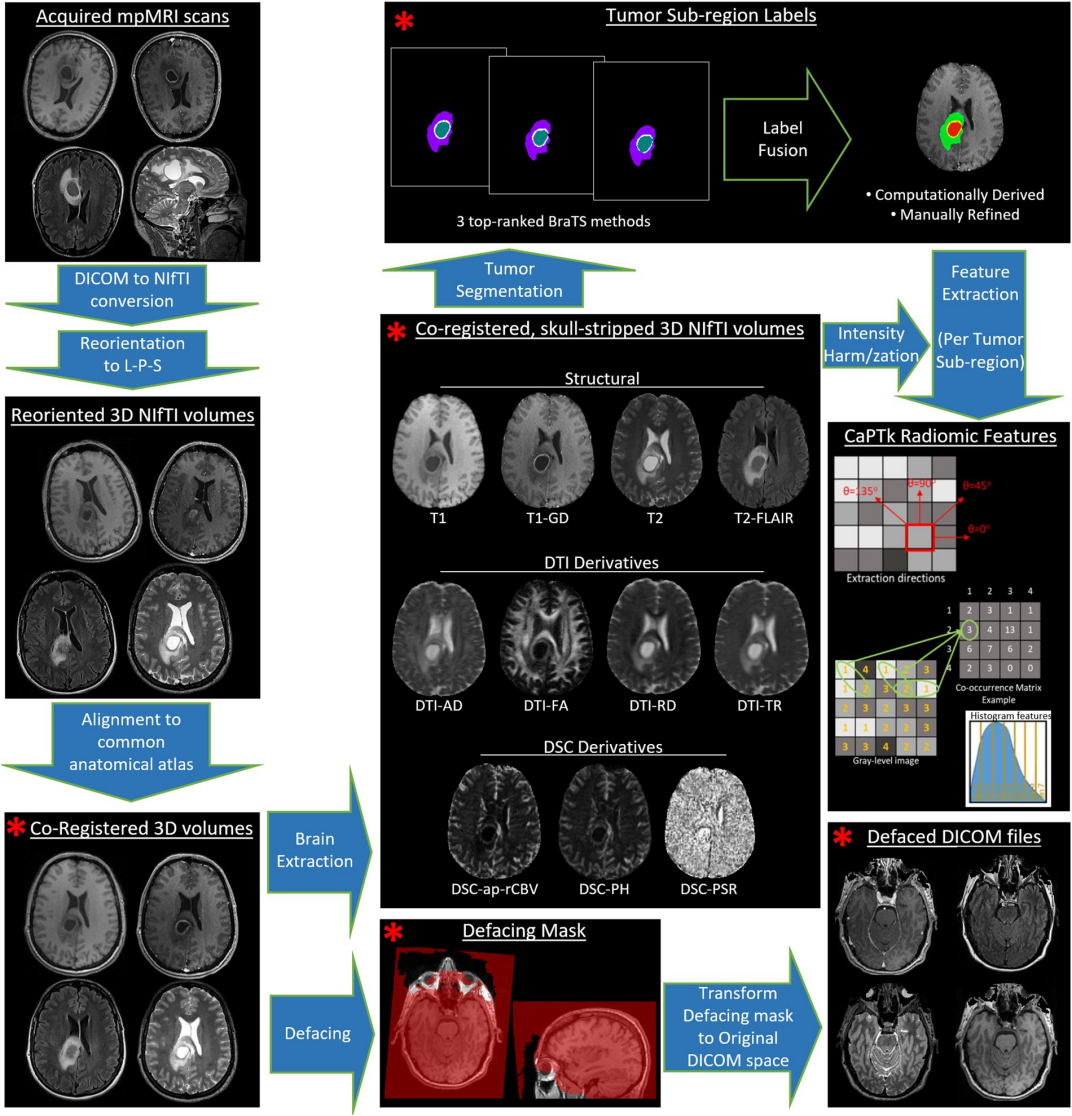
Schematic representation of the harmonized pre-processing pipeline applied to all the UPenn-GBM imaging data.

All DICOM scans were converted to the Neuroimaging Informatics Technology Initiative (NIfTI)^49^ file format to facilitate computational analysis, following the well-accepted pre-processing protocol of the International Brain Tumor Segmentation (BraTS) challenge^15–18,33,50^. Specifically, all mpMRI volumes were reoriented to the left-posterior-superior (LPS) coordinate system, and the T1-Gd scan of each patient was rigidly (6 degrees of freedom) registered and resampled to an isotropic resolution of 1 *mm*^3^ based on a common anatomical atlas, namely SRI^51^. The remaining scans (i.e., T1, T2, T2-FLAIR) of each patient were then rigidly co-registered to this resampled T1-Gd scan by first obtaining the rigid transformation matrix to T1-Gd, then combining with the transformation matrix from T1-Gd to the SRI atlas, and resampling. For all the image registrations we used the ‘Greedy’ (https://github.com/pyushkevich/greedy, hash: 1a871c1, Last accessed: 27/May/2020) tool^52^, which is a central processing unit (CPU)-based C++ implementation of the greedy diffeomorphic registration algorithm^53^. We further note that use of any non-parametric, non-uniform intensity normalization algorithm^54–56^ to correct for intensity non-uniformities caused by the inhomogeneity of the scanner’s magnetic field during image acquisition, obliterates the T2-FLAIR signal, as it has been previously reported^16^. Thus, taking this into consideration, we intentionally apply the N4 bias field correction approach^55^ in all scans temporarily to facilitate an improved registration of all scans to the common anatomical atlas. Once we obtain the transformation matrices for all the scans, then we apply these transformations to the non-bias corrected images. A schematic summary of the preprocessing protocol applied to all the UPenn-GBM data collection can be found at Fig. 2.

### Image De-facing

All co-registered scans were defaced, using the ‘mri_deface’ tool (https://surfer.nmr.mgh.harvard.edu/fswiki/mri_deface)^57^. Specifically, the defacing mask is first obtained for the co-registered native T1-weighted scan, the sequence with which ‘mri_deface’ is designed to work. The defacing masks for all cases were visually checked and approximately 10% of the masks were manually refined. The defacing mask was then applied to all co-registered structural MRI scans (i.e., T1-Gd, T1, T2, T2-FLAIR). These defaced data are available as the “unstripped-structural” scans in the UPenn-GBM collection, and can be used for studies requiring the skull, such as the evaluation of automatic brain extraction methods^58^. Furthermore, the defacing masks for each subject were transferred back to the original space, consistent with the MRI acquisition and DICOM format data, by applying the inverse transformation through the ‘Greedy’ tool^52^. These masks were then applied to the scans in the original space, and the resultant de-faced images were written back to the DICOM format using CaPTk^35,59,60^ and made available in the UPenn-GBM collection. No defacing masks were applied to the DSC and the DTI scans since facial information is not included in the originally acquired scans.

### Brain extraction

Further to defacing all scans, we also extracted the brain using a routine process in neuroimaging called skull-stripping (also known as brain extraction). This process focuses on generating a brain mask to remove all non-brain tissue from the image (including neck, fat, eyeballs, and skull), to enable further computational analyses. Notably, for the brain extraction we used an in-house deep learning based approach, namely the Brain Mask Generator (BrainMaGe)^58^ (https://github.com/CBICA/BrainMaGe), which has been explicitly developed to address brain scans in presence of diffuse glioma, and takes into consideration the brain shape as a prior, hence being agnostic to the sequence/modality input. Once the brain mask is generated, reviewed, and approved for a single sequence, it is then applied to all co-registered scans to obtain the skull-stripped images included in the UPenn-GBM collection.

### Tumor sub-region segmentation

**Fig. 3:**
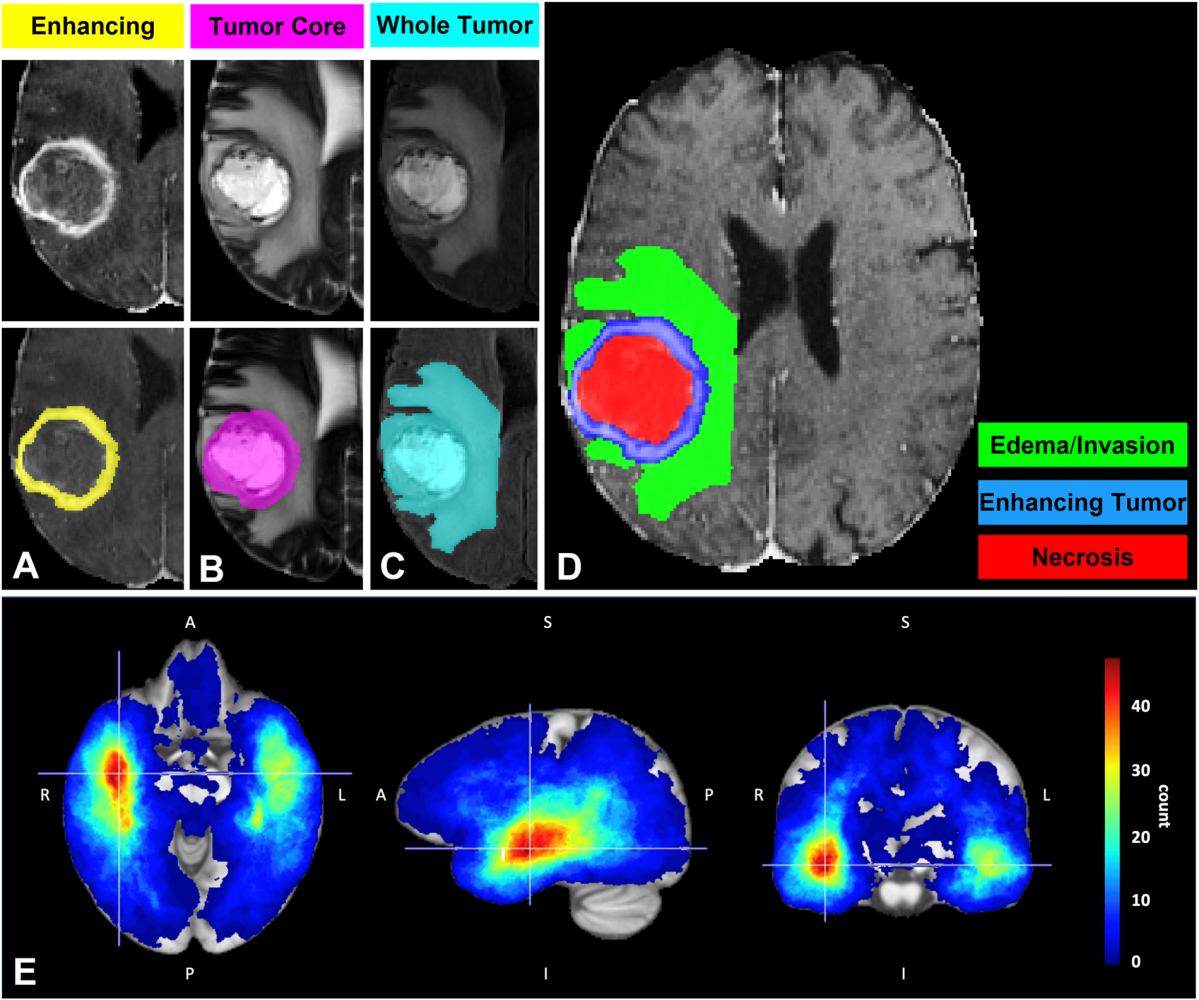
Glioma sub-region labeling (**A**–**D**) and the overall tumor distribution atlas of the UPenn-GBM data collection (**E**). Sub-figures A-D depict an example visual representation of the segmented glioma sub-regions labels superimposed on different MRI scans. (**A**) the enhancing tumor (ET - yellow) superimposed on a T1-Gd scan, surrounding the cystic/necrotic components of the tumor core; (**B**) the tumor core (TC–magenta) superimposed on a T2 scan, highlighting the potentially resectable tumor; (**C**) the whole tumor (WT - cyan) superimposed on a T2-FLAIR scan, showing all the abnormal tissue; (**D**) depicts the WT discretised in the independent histologically-distinct tumor sub-region labels: enhancing tumor core (blue), necrotic/cystic core (red), and peritumoral edematous/infiltrated tissue (green). (**E**) depicts the spatial distribution of the TC from the complete set of the UPenn-GBM collection’s pre-operative scans.

Finally, the histologically distinct tumor sub-regions were segmented using a fully-automatic approach, based on the label fusion of a few deep learning algorithms that have been top-ranked in the BraTS challenge^15–18,33,50^. Specifically, the segmentation labels of the enhancing tumor (ET), the necrotic tumor core (NCR), and the peritumoral edematous/infiltrated tissue (ED) are considered. Radiographically, the ET and NCR parts are defined by hyper-intense and hypo-intense areas, respectively, on T1-Gd compared with T1, but also compared with normal-appearing white matter. The NCR regions describe non-enhancing or faintly enhancing tumor core components, as well as transitional/pre-necrotic and necrotic regions that belong to the non-enhancing part of the bulk tumor, and are typically surgically resected together with the ET. Finally, the ED region is defined by the abnormal hyperintense signal envelope on the T2-FLAIR volumes. A visual example of segmented tumor sub-regions can be found in Fig. 3.

The patient’s co-registered and skull-stripped structural mpMRI (i.e., T1, T1-Gd, T2, T2-FLAIR) were used as the input to each of the segmentation algorithms (i.e., DeepMedic^61^, DeepSCAN^62^, and nnUNet^63^) that partition the patient’s brain into the three aforementioned tumor sub-region labels, including a single label for everything else. Subsequently, we applied the STAPLE label fusion technique^64^ to appropriately combine the results of the three algorithms and overcome errors of individual methods. Furthermore, the tumor segmentation labels of a subset of 232 subjects were evaluated by S.M. and M.B. and manually refined when needed.

### Perfusion and diffusion derivative volumes

We have used the acquired DTI volumes to extract commonly used derivative diffusion measurements that can be associated with the tissue microstructure and density^65^, in the form of individual volumes, comprising the (i) tensor’s trace (DTI-TR), (ii) axial diffusivity (DTI-AD), (iii) radial diffusivity (DTI-RD), and (iv) fractional anisotropy (DTI-FA). Furthermore, the DSC-MRI volumes were used to extract parametric maps of isolated measurements summarizing the complete dynamic 4D perfusion signal into a single 3D volume. These maps comprise the i) peak height (DSC-PH), ii) percentage signal recovery (DSC-PSR), and an automated proxy to the relative cerebral blood volume (DSC-ap-rCBV)^11,66–68^. Note that both the DTI and the DSC derivative maps can all be used as individual imaging volumes for further analyses. The UPenn-GBM data also includes the DTI and DSC derivative volumes co-registered with the skull-stripped structural images to enable further computational studies.

### Feature extraction

Following the definition of the distinct tumor sub-regions, all mpMRI sequences were analyzed to extract relevant comprehensive quantitative imaging phenomic (QIP) features from each of the corresponding sub-regions. Such features have been extensively used for the development of predictive models for diagnostic, planning, and prognostic purposes, as well as to characterize CNS tumors comprehensively, and provide critical information about various biological processes within the tumor microenvironment, as well as associations with underlying cancer molecular characteristics^11,12,14,39,68–107^.

We have specifically extracted 145 features for each annotated sub-region and from each MRI sequence separately using the Cancer Imaging Phenomics Toolkit (CaPTk, www.cbica.upenn.edu/captk)^35,59,60,108,109^, which has been extensively used in radiomic analysis studies^16,58,69,97,109^. The exact features extracted from CaPTk, in compliance with the Image Biomarker Standardisation Initiative (IBSI)^34,110^, include five primary feature groups: i) intensity-based features, ii) histogram-related, and iii) volumetric measurements, iv) morphological parameters, and v) textural descriptors. The intensity-based features include first-order statistics (e.g., mean, median, maximum, minimum, standard deviation, skewness, kurtosis) capturing information of the overall intensity distribution profile within each sub-region within a given image/scan. Additional characteristics are provided by histogram-related measures, which describe the range and distribution of image grey-level intensity levels. Volumetric parameters capture shape information in morphologic metrics such as elongation, perimeter, principal component axes, and area or volume for two- or three-dimensional data, respectively. Last, textural descriptors include a wide range of indices describing the local variation and spatial dependence of image intensities (based on grey-level co-occurrence (GLCM)^111^, grey-level run-length (GLRLM)^112–116^, gray-level-size zone (GLSZM)^113–115,117^, and neighborhood gray-tone difference (NGTDM) matrices)^118^, as well as local binary patterns^119^, which characterize intrinsic periodic texture structures that repeat over multiple image scales.

CaPTk ships with a default parameter file for feature calculations, which, however, can be customized by the user based both on image protocol specifications (e.g., slice thickness, pixel/voxel resolution, image reconstruction filters), but also allows for further optimization of these parameters based on feature associations with specific endpoints of interest, such as molecular markers, clinical outcomes, treatment responses, and other patient outcomes, to further boost feature performance. CaPTk’s default parameterization values were the ones we considered to obtain the features that we are providing. We provide these extracted radiomic features on an ‘as-is’ basis, while making no claim for their superiority or their biological significance. These are included here to facilitate research on their association with molecular markers, clinical outcomes, treatment responses, and other endpoints, by researchers without sufficient computational background to extract such features. The list of the radiomic features extracted, as well as the specific parameterization file for extracting them through CaPTk, are made available and can downloaded from the TCIA repository^37^.

## Data Records

All the data described here as the “UPenn-GBM” collection^37^, are available from the publicly available repository of The Cancer Imaging Archive (TCIA)^31^ at: 10.7937/TCIA.709X-DN49. Data availability per subject can also be found and downloaded from the TCIA repository^37^.

## Technical Validation

### Clinical data & molecular characterizations

All clinical and molecular characteristics of the subjects included in the “UPenn-GBM” collection were obtained retrospectively from clinical records (e.g., radiology & pathology reports), that were used for patient management. No additional validation of these raw clinical data was conducted as part of the “UPenn-GBM” release.

### Image processing steps

All the image processing related steps were manually reviewed, and either approved, or corrected as deemed necessary. However, it is important to note that there was a high level of uncertainty reported by radiologists, radiation oncologists, surgeons, and imaging scientists as to the exact boundaries between the various tissues assessed, most notably in the tumor labels, where visual assessment is always ambiguous.

#### Image pre-processing

The image pre-processing pipeline included a manual assessment for sufficient quality at various steps, as indicated in Fig. 2. Specifically, after the registration of all mpMRI volumes to the common anatomical atlas, all scans were manually reviewed for misalignment and corrected through ITK-SNAP^120,121^ when necessary.

#### Image de-facing

The derived defacing masks for all cases were visually checked for quality, and approximately 10% of them were manually refined as needed.

#### Brain extraction & tumor segmentation

The automatically derived brain masks and tumor segmentation labels for all the included cases were visually checked for quality and manually refined as needed, prior to proceeding with further analysis. Manual refinements that were applied in the computer-aided segmentation labels comprise: i) obvious under- or over-segmentation of regions (brain/ET/NCR/ED), ii) voxels classified as ED within the tumor core, iii) unclassified voxels (i.e., holes) within the tumor core, iv) voxels classified as NCR outside the tumor core. Note that in line with the protocol followed by BraTS, during the manual corrections only peritumoral ED was considered, and contralateral, as well as any periventricular ED was removed, unless it was a contiguous area with the peritumoral ED. The rationale for this is that contralateral and periventricular white matter hyper-intensities might be considered pre-existing conditions, and/or related to small vessel ischemic disease, especially in older patients.

### Perfusion derivatives

The DSC scans are obtained by preloading half of the total contrast agent to reduce the effect of contrast agent leakage, followed by the second half of the total contrast volume. The raw signal time curve was inspected to confirm proper bolus administration, as the data must have sufficient time before and after the signal drop to calculate the derivative images including PH, PSR, and ap-RCBV^11,66–68^. Any images that did not meet these criteria were excluded from further analysis.

### Diffusion derivatives

Diffusion tensor derivatives were manually inspected for coverage of the entire brain and their co-registration to the structural MRI scans. Fractional anisotropy values were required to be real values between 0 and 1. Unweighted (b = 0) images were required to be positive, due to numerical problems that arise when fitting a tensor in a voxel with an unweighted value of 0. Any voxels that did not meet these requirements were excluded from further analysis.

### Feature extraction

Considering the mathematical formulation of these features, it is possible for a division by zero to occur (lack of heterogeneity or very small number of voxels). In CaPTk, we return “not a number” as the result of these features to enable the user to make subsequent downstream analyses more coherent based on the entire population. We acknowledge this could be provided as “inf” instead, but we are providing this as “NaN” to have parity between various programming languages and processing protocols.

## Usage Notes

Potential use cases of the hereby presented UPenn-GBM data collection could be influenced by and aligned with previous findings utilizing subsets of the data collection. Specifically, cases that have been included in previously example published studies, comprise 63 cases used in a study to predict the location of tumor recurrence from pre-operative baseline MRI scans^95^, 120 cases used in a study predicting the molecular subtype of glioblastoma and performing an imaging-based patient prognostic stratification^97^, 98 cases used in a study predicting patient overall survival utilizing only routine structural MRI scans^69^, 173 cases used in the International Brain Tumor Segmentation (BraTS) challenge^18^, and in the Federated Tumor Segmentation (FeTS) challenge^122^, 60 cases used in a study distinguishing true progressive disease from pseudoprogression^39^, 250 cases used in a study identifying imaging subtypes offering prognostic value beyond IDH^99^, and 86 cases used in a study designing a brain extraction method explicitly designed for brain glioma MRI scans^58^.

## Data Availability

In line with the scientific data principles of Findability, Accessibility, Interoperability, and Reusability (FAIR)^123^, the tools used throughout the generation of these data are publicly available. Specifically, we have used the Insight Toolkit (ITK)^124^ to convert the raw DICOM files to the NIfTI file format^49^, and when this mechanism did not work we have used the dcm2niix software (version 1.0.20200331)^125^. All image registrations were performed using the ‘Greedy’ registration algorithm (https://github.com/pyushkevich/greedy)^52^, a CPU-based C++ implementation of the greedy diffeomorphic registration algorithm^53^. ‘Greedy’ is integrated into the ITK-SNAP segmentation software^120,121^ (https://www.itksnap.org/, version: 3.8.0, last accessed: 27/May/2020), as well as into the Cancer Imaging Phenomics Toolkit (CaPTk)^35,59,60^ (www.cbica.upenn.edu/captk, version: 1.8.1, last accessed: 11/February/2021). For the defacing of the acquired scans we used the “mri_deface” tool (https://surfer.nmr.mgh.harvard.edu/fswiki/mri_deface), and for the brain extraction we used BrainMaGe version 1.0.4^58^ (https://github.com/CBICA/BrainMaGe). In addition, the CaPTk platform^35,59,60,108,109^, version 1.8.1, was used for all the preprocessing steps, as well as for obtaining the perfusion derivatives, and generating the output DICOM files (https://cbica.github.io/CaPTk/ht_utilities.html) after defacing of the data. The implementation producing the diffusion derivatives will be available in CaPTk v.1.9.0. CaPTk’s source code and binary executables are publicly available for multiple operating systems through its official GitHub repository (https://github.com/CBICA/CaPTk). The implementation and configuration of the pre-trained segmentation models used in this study can be found in the GitHub page of the Federated Tumor Segmentation (FeTS) platform (https://github.com/CBICA/FeTS). Finally, ITK-SNAP^120,121^ version 3.8.0 was used for all manual annotation refinements.
